# Maturation- and aging-related differences in electrophysiological correlates of error detection and error awareness

**DOI:** 10.1016/j.neuropsychologia.2020.107476

**Published:** 2020-06

**Authors:** Franka Thurm, Shu-Chen Li, Dorothea Hämmerer

**Affiliations:** a Chair of Lifespan Developmental Neuroscience, Faculty of Psychology, TU Dresden, Dresden, Germany; b Centre for Tactile Internet with Human-in-the-Loop (CeTI), TU Dresden, Dresden, Germany; c German Center for Neurodegenerative Diseases (DZNE), Magdeburg, Germany; d Institute of Cognitive Neurology and Dementia Research, Otto-von-Guericke-University Magdeburg, Magdeburg, Germany; e Center for Behavioral Brain Sciences, Magdeburg, Germany; f Institute of Cognitive Neuroscience, University College London, London, UK; g Wellcome Centre for Human Neuroimaging, London, UK

**Keywords:** Performance monitoring, Lifespan development, Error-related negativity, Error positivity

## Abstract

The error-related negativity (ERN/Ne) as well as the early and late error positivity (Pe) are electrophysiological correlates known to reflect error detection and error awareness. Despite much evidence on age differences in mastering response conflicts, the development and the functional distinctiveness of these components across the lifespan is still unclear. Here we investigated maturation- and aging-related differences in the ERN/Ne, the early and late Pe during a response conflict task in a lifespan sample that included 45 children, 42 adolescents, 39 younger and 34 older adults. Lifespan age differences were characterized by marked declines of all three components in older age, whereas clear maturation effects from childhood to adolescence were only observed for error detection reflected in the ERN/Ne component. Furthermore, using regression analyses, we examined functional relationships of the error monitoring components to behavioral indicators of task performance. Across all age groups, both the ERN/Ne and the early Pe were related to response accuracy, but only the early Pe was further associated with performance in a covariate task indicative of perceptual processing and attention capacities. Our results suggest that the ERN/Ne, the early and late Pe reflect distinct but complementary processes of error monitoring across the lifespan.

## Highlights

• ERP correlates of error monitoring were investigated from childhood to old age.
• Aging affected ERP components of error detection and awareness.
• Maturation from childhood to adolescence only affected error detection (ERN/Ne).
• Only the early Pe was related to perceptual processing and attention allocation.
• ERN/Ne and Pe components distinct but complementary processes of error monitoring.

## 1 Introduction

“Errare humanum est, sed in errare perseverare diabolicum” or “to err is human, but to persist in error is diabolical” (L. A. Seneca, Epistulae morales ad Lucilium, 62–65 A.D.).

Since the ancient Romans, the ability to avoid the repetition of errors has been considered as a crucial aspect of human behavior. Avoiding the repetition of errors in changing or uncertain environments requires a performance monitoring system that allows for rapid detection and evaluation of errors or error-prone situations. Indeed, electroencephalography (EEG) research focusing on event-related potential (ERP) suggests that the human performance monitoring continuously monitors error in sequential stages. More specifically, ERPs reflecting rapid error detection are directly followed by potentials that vary with error awareness, which are then followed by potentials reflecting an evaluation of the affective significance (i.e., valence) of detected errors. The earlier process of error detection is presumably relatively implicit, whereas the later processes of error awareness and evaluation are considered explicit (Steinhauser and Yeung, 2010; see Ullsperger et al., 2014, for a review; Van Veen and Carter, 2002). The ability to successfully master tasks that require performance monitoring (i.e., situations prone to errors of response execution) varies considerably across the human lifespan. Indeed, age differences in efficient performance monitoring could be expected given maturation- and aging-related changes in its underlying brain substrates, such as the anterior cingulate cortex (ACC) and the prefrontal cortex (PFC), as well as in neuromodulatory processes in the dopamine (DA) system (see Hämmerer et al., 2014 for review). However, a systematic investigation of lifespan age differences in neural correlates reflecting the sequential stages of performance monitoring is so far lacking. Thus, the current study aimed to gain insights into the maturation and aging of ERPs associated with the early process of error detection (i.e., ERN/Ne; Falkenstein, Hohnsbein, Hoormann and Blanke, 1990, 1991) and later processes of error awareness and evaluation (i.e., early and late Pe; Falkenstein, Hohnsbein and Hoormann, 1994; Nieuwenhuis et al., 2001). To do so, we compared these different aspects of performance monitoring in children, adolescents, younger and older adults during a continuous performance Go-Nogo task (cf. Hämmerer et al., 2010). We validated the presumed cognitive processes reflected in these ERPs by assessing their independence from each other as well as their relationship to behavioral indicators relevant for error detection or error awareness. In the following, we review existing evidence on the sequential stages of performance monitoring and related ERP components before outlining how these stages might differ in the course of lifespan development from childhood to old age.

Electrophysiological and computational modeling studies have provided ample evidence suggesting that erroneous responses in speeded choice reaction time tasks elicit a uniform temporal sequence of ERPs across individuals, with an early frontocentral negative deflection (ERN/Ne) peaking around 50–100 ms after response, directly followed by an early frontocentral positivity (early Pe) and a later centroparietal positive deflection (late Pe) peaking around 200–500 ms after the error occurred (cf. Ullsperger et al., 2014 for review of the temporal dynamics of EEG correlates of performance monitoring; Ullsperger and von Cramon, 2006). In this sequence, the ERN/Ne reflects error detection (Di Gregorio, Steinhauser and Maier, 2016; Holroyd and Coles, 2002; Nieuwenhuis et al., 2001; Yeung et al., 2004). In contrast, the late Pe component is assumed to reflect explicit error awareness i.e., the output of the decision process whether or not an error has occurred (Endrass et al., 2012; Endrass et al., 2007; Hughes and Yeung, 2011), whereas the early Pe is hypothesized to rather code the accumulated error evidence and therefore to provide the input into the decision process similar to the ERN/Ne (Steinhauser and Yeung, 2010; Wessel et al., 2011). In line with these assumptions about the functional distinctions of the ERN/Ne and the Pe components, source modeling (Herrmann et al., 2004; Van Veen and Carter, 2002) and combined EEG-fMRI studies (Debener et al., 2005) indicate that error-related activation in the caudal ACC is associated with both the ERN/Ne and the early Pe component, whereas the late Pe seems to be generated in more rostral regions of the ACC and the superior parietal cortex. Given the close temporal and regional proximity of the ERN/Ne and the early Pe and given that both ERP components rather reflect implicit stages of error monitoring, it is still debated whether the ERN/Ne and the early Pe reflect dissociable or rather common processes of the performance monitoring system (Van Veen and Carter, 2002). Assessing lifespan age differences in ERP components associated with error detection and awareness and their relationships with performance measures may provide further insights into potential functional distinctions and similarities of the three components.

Thus far, maturation- and aging-related differences in ERN/Ne and the subsequent early versus late Pe have not been directly compared in one lifespan sample covering children, adolescents, younger and older adults. Based on previous results of mostly separate developmental and aging studies, we expected the ERN/Ne to show a curvilinear pattern across the lifespan, suggesting ERN/Ne amplitudes being attenuated in children (e.g., Davies et al., 2004; Hogan et al., 2005; Ladouceur et al., 2007; Overbye et al., 2019; Santesso and Segalowitz, 2008; Santesso et al., 2006; Wiersema, van der Meere and Roeyers, 2007) and older adults (e.g., Falkenstein et al., 2001; Mathewson et al., 2005; Themanson et al., 2006; but Eppinger et al., 2008; Thurm et al., 2013). Evidence on the development of the early and late Pe components, related respectively to implicit error monitoring and explicit error awareness, seems less consistent. Variations in the characteristics (i.e., time window and electrode sites) of Pe and the lack of differentiation between the early and late Pe component in most studies may have contributed to the inconsistencies of findings. The majority of developmental studies reported comparable Pe components (resembling the late Pe) in children, young adolescents, and young adults (Davies et al., 2004; Santesso and Segalowitz, 2008; Wiersema et al., 2007), whereas others reported smaller Pe amplitudes in children (Santesso et al., 2006) or larger Pe amplitudes in older adolescents (Ladouceur et al., 2007) compared to young adults. Limited evidence from studies that investigated the Pe in aging populations indicates an attenuated Pe in older compared to young adults (Band and Kok, 2000; Clawson et al., 2017; Mathewson et al., 2005; Niessen et al., 2017). Based on these prior findings, we expect the late Pe to be most importantly affected by aging-related decline but to be less affected by maturation processes during childhood and adolescence. In contrast, given current views that the early Pe might be more closely related to error detection processes that also underlie the ERN/Ne (Endrass et al., 2007), we expect an inverted U-shaped pattern for the early Pe that is more similar to the lifespan age differences of the ERN/Ne than the late Pe.

## 2 Methods

We re-analyzed data from an earlier study on lifespan development of response conflict monitoring (Hämmerer et al., 2010). Different from the previous study, which focused only on stimulus-locked brain potentials, the current analyses focus on response-locked brain potentials (particularly early and late Pe) related to error detection and awareness.

### 2.1 Participants

Altogether, 160 subjects were included, covering four age groups across the lifespan: 45 children (CH; 9–10 years; 23 males), 42 adolescents (TE; 13–15 years, 23 males), 39 younger adults (YA; 21–28 years, 21 males), and 34 older adults (OA; 66–76 years, 18 males). Previous analysis of this sample revealed moderate effect sizes (inter-class correlation coefficients) of 0.42–0.69 for age group differences in response-locked ERPs related to performance monitoring processes (Hämmerer et al., 2010). The sample was therefore considered sufficient for stimulus-locked analysis of ERPs related to error detection and error awareness.

All subjects were right-handed according to Edinburgh Handedness Inventory (lateralization quotient (LQ) > 80; Oldfield, 1971). Following EEG data preprocessing and prior to statistical analyses, subjects were excluded from the initial sample when both target channels for the early Pe (FCz, Cz) or late Pe (CPz, Pz) had to be removed during artifact correction or when fewer than two trials per condition remained after artifact correction (cf. Olvet and Hajcak, 2009). Sample characteristics are provided in Table 1. Each participant or parent of the participant gave written informed consent. The study was approved by the Ethics Committee of the Max Planck Institute for Human Development, Berlin, Germany. Participants were paid 10€ for the first, and 7€ for every following hour of the experiment for compensation.

**Table 1:** Lifespan sample characteristics and mean behavioral performance in the cognitive covariate tasks.

Variable	Children		Adolescents		Younger Adults		Older Adults	
	(9–11 years)		(13–15 years)		(21–28 years)		(66–76 years)	
n	45		42		39		34	
Age (years)	10.2	(0.7)	14.4	(0.7)	24.2	(2.0)	70.8	(2.9)
Females (%)	49		45		46		47	
SAW correct (%) a	4.6	(4.3)	12.6	(6.2)	20.5	(5.2)	25.9	(5.3)
DS correct (no.) a	35.6	(7.7)	51.7	(9.9)	62.0	(11.1)	45.1	(8.1)
IDP RT median (ms) a	3090.2	(624.6)	2205.2	(390.9)	2085.7	(411.8)	3558.0	(581.1)
IDP RT variability (ms) a	1089.3	(303.9)	844.4	(294.0)	729.3	(250.7)	1267.6	(453.0)
Early IT correct (no.) a	7.3	(1.0)	7.8	(0.9)	8.1	(0.9)	7.3	(1.0)

*Note.* If not otherwise specified, values indicate means (*M*) with standard deviations (*SD*) in parenthesis. DS – Digit-Symbol-Substitution Test; IDP – Identical Pictures Test; IT – Inspection Time Test RT – reaction time; SAW – Spot a Word Test.

a Significant age group effects with p < 0.05.

### 2.2 Task and procedure

Subjects performed a version of the Continuous Performance Task (CPT-AX; Hämmerer et al., 2010), which was modified to be applied with all four age groups, including children and older adults. In a nutshell, altogether 930 squares of 12 of different colors were presented successively on a white screen. Subjects were instructed to respond as fast as possible by pressing a button with their right index finger only when the blue square (Cue) was followed by the yellow square (Target). Responses to the remaining 10 colored squares (Non-targets) had to be inhibited. The task included 270 Go pairs (58%; Cue and Target in succession) and 195 Nogo pairs, including 65 NogoPrime pairs (14%; Cue and Non-target in succession). Based on pilot data assessments, the response time deadline was adapted for each age group with 500 ms after stimulus onset for adolescents and younger adults and 650 ms after stimulus onset for children and older adults.

Prior to the experiment, subjects underwent a short training with a sequence of 20 stimuli. Subsequently, the total number of 930 trials was presented in 5 blocks of 186 trials each (approx. 35 min task duration in total). After each block, subjects received feedback about their task performance on the computer screen (i.e., number of correct responses and number of timeouts) and could take a short self-timed break. Before continuing with the next block, subjects were again instructed to respond as fast and as accurate as possible.

### 2.3 Cognitive covariate assessment

We assessed two components of lifespan cognition (Baltes, 1987; Lindenberger, 2001) prior to the EEG acquisition in a separate appointment. A version of the digit symbol substitution test (Wechsler, 1981) assessed perceptual speed and served as a measure of fluid cognitive abilities, which reflect more basic cognitive mechanics and are more affected by development and aging across the lifespan. The spot a word test (Lindenberger and Baltes, 1997) assessed verbal knowledge as an indicator of crystalized abilities, which accumulate with knowledge acquisition and experience in the course of the lifespan and, hence, improve with increasing age of healthy subjects (Li et al., 2004). The test results for each age group, which are consistent with lifespan age gradients of fluid and crystalized intelligence measures in a larger and representative sample (Li et al., 2004), are summarized in Table 1.

In addition, computerized versions of the inspection time task (Kranzler and Jensen, 1989; Vickers and Smith, 1986) and the identical pictures task (Lindenberger and Baltes, 1997) were also assessed in order to measure early (implicit) stimulus processing and overall speed of processing, respectively. During the inspection time task, subjects were asked to identify the longer of two lines that were presented only briefly on the screen (with 75 Hz refresh rate) and then covered by a mask. Altogether, 12 different presentation durations (i.e., inspection times) were applied to assess maturation- and aging-related differences in implicit stimulus processing time as an indirect measure of early attention allocation. Especially the early (i.e., short) inspection time has previously been associated with ERPs related to early visual processing and early attention allocation such as the P2 (Caryl, 1994; Zhang et al., 1989). Therefore, early inspection time was computed as the mean response accuracy across 10 repetitions in trials with 27, 40, 54, 67, or 80 ms inspection times. For the identical pictures test, subjects had to match as many simple schematic black-and-white pictures as possible within 80 s. Responses were given by pressing a button on a response pad and reaction times (RT) were recorded for each trial.

### 2.4 EEG data acquisition

EEG was recorded continuously from 64 Ag/AgCl passive electrodes using BrainVision Recorder (BrainAmp DC amplifiers, Brain Products GmbH, Gilching, Germany). EEG electrodes were placed according to the 10-10 system using an elastic cap (Braincap, BrainVision) with the right mastoid as reference and the ground positioned over the forehead. Additionally, the horizontal and vertical electrooculogram (EOG) was recorded. The sampling rate was set to 1000 Hz with a 0.01–250 Hz bandpass hardware filter. EEG recordings were referenced online to the right mastoid. The ground was positioned above the forehead. Impedances were kept below 5 kΩ.

### 2.5 Data analysis

#### 2.5.1 Behavioral data analysis

Trials were classified as correct responses when subjects responded to the Target following the Cue (Go trials) and as prime-based errors when subjects responded to a Non-target following the Cue (NogoPrime trials). Trials with responses to the Target following a Non-target, with responses to a Non-target following another Non-target or with responses to the Cue were not included into the analysis. Reaction times below 100 ms and larger than 2000 ms and, subsequently, reaction times slower or faster than 3.5 standard deviations of the mean reaction time within each age group were excluded. For statistical analysis, response speed was quantified as the median reaction time for correct Go trials (cf. Rabbitt, 1966). RT trial-to-trial variability was computed as the standard deviation of reaction times across all correct Go trials to correct for non-normal distribution (cf. Papenberg et al., 2013). Performance accuracy (i.e., error rate) was quantified as the percentage of NogoPrime responses out of altogether 65 NogoPrime trials.

#### 2.5.2 ERP analysis

Preprocessing of the recorded data and the extraction of ERP components was carried out using EEGLAB (Delorme and Makeig, 2004; https://sccn.ucsd.edu/eeglab/) and Fieldtrip toolboxes (http://www.ru.nl/fcdonders/fieldtrip) in a Matlab environment (https://www.mathworks.com/). In accordance with Hämmerer et al. (2010), the data were re-referenced offline to an averaged mastoid reference and segmented into epochs of 1.5 s before and 2.5 s after stimulus onset. Epochs or channels with marked muscular artifacts or saturated recordings were excluded manually. An average of 12% of the trials had to be removed from the EEG data (CH: 19.25%, TE: 12.56%, YA: 8.41%, OA: 8.10%). Subsequently, ocular and additional muscular components were removed using independent component analysis (ICA) decomposition in EEGLAB (Delorme and Makeig, 2004). Recombined data were bandpass-filtered in the range of 0.5–25 Hz and epoched 100 ms before and 1000 ms after correct and erroneous responses with a 100 ms pre-response baseline correction. Go trials (correct responses) and NogoPrime trials (prime-based errors) were averaged for each electrode and subject. The Pe was computed as the difference waveform between erroneous NogoPrime trials and correct Go trials (cf. Steinhauser and Yeung, 2010). Individual peaks (i.e., positive maximums) were detected in the time window of 100–250 ms post-response at electrode sites FCz and Cz for the early Pe component and 250–400 ms post-response at electrode sites CPz and Pz for the late Pe component (Steinhauser and Yeung, 2010; Ullsperger et al., 2014; Van Veen and Carter, 2002). Mean amplitudes for both Pe components were then computed in the time window of ±50 ms centered at the individual peak. The Ne/ERN difference waveform was computed analogously with a time window for peak detection (i.e., negative maximum) of 0–100 ms post-response at electrode sites FCz and Cz. ERP data were further averaged across both respective electrode sites (FCz and Cz for ERN/Ne and early Pe and CPz and Pz for the late Pe; cf. e.g., Endrass et al., 2007) to control for potential age group differences in ERP topography (cf. Hämmerer et al., 2010).

#### 2.5.3 Statistical analysis

Statistical analysis was performed using IBM SPSS Statistics 24.0 (http://www-01.ibm.com/software/de/analytics/spss/). Sociodemographic and behavioral task performance measures were compared between the four groups using univariate analysis of variance (ANOVA) models. Differences in gender distribution between age groups were assessed using the Pearson Chi-squared (*Χ*^*2*^) test. For statistical comparison of ERP components between age groups, separate univariate ANOVA models and planned contrasts (linear, quadratic) were computed for the ERN/Ne, early Pe, and late Pe components and for amplitudes and latencies separately with age group as between-subject factor. Follow-up planned contrasts were conducted in three steps to further compare the ERP components: (1) in the young adults (YA; baseline) versus the developmental (CH, TE) and aging samples (OA) (contrasts: [-1 0 1 0], [0–1 1 0], [0 0 1 -1]), (2) between both developmental samples (CH, TE) (contrast: [-1 1 0 0]), and (3) between the children (CH) and older adult samples (OA) (contrast: [-1 0 0 1]).

Separate linear hierarchical regression models were conducted across all four age groups to further investigate potential relations between the amplitudes of the three ERP components (entered as outcome variables) and (1) behavioral performance during the CPT-AX task, namely median RT (Go trials), accuracy (NogoPrime errors), and intra-individual RT variability (i.e., trial-by-trial standard deviation of RTs), and (2) behavioral performance in the covariate inspection time task (i.e., early visual processing accuracy and attention allocation). In order to adjust for intercept differences between age groups in the regression models (i.e., to enable regression analysis across all age groups), dummy coded age group variables were entered first into all regression models. The purpose of the age group dummy variables was to account for age group main effects and related intercept differences (Papenberg et al., 2013). The dummy variables were then followed by a regressor of no interest, continuous age (*z*-scored) to account for variability in age within age groups. Next, we entered amplitudes of ERP components occurring prior to the ERP component which was investigated as outcome variable into the hierarchical regression models to control for possible interrelations between successive ERP components according to their temporal order (i.e., only the ERN/Ne was entered into the early Pe models and both the ERN/Ne and the early Pe were entered into the late Pe models). Since the ERN/Ne precedes both Pe components, no further ERP component was entered into the ERN/Ne regression models. In addition, to directly investigate potential rebound effects of the ERN/Ne on the Pe components, two linear hierarchical regression models with both Pe components as dependent variables and the ERN/Ne as predictor were conducted. Age dummy variables and continuous age were entered into these models as well as previously described. Median RT was entered next into all models examining RT variability as regressor of no interest to account for dependence between response variability and response speed.

Bivariate relations between continuous variables were computed using Pearson's *r* correlation coefficient. Partial eta-squared (*η*^*2*^) was computed as indicator for effect sizes of ANOVA analyses and planned contrasts. For the regression models, effect sizes of the regressors were compared based on their respective beta scores. Significance threshold was set to alpha (*α*) ≤ 0.05.

## 3 Results

### 3.1 Lifespan differences in behavioral CPT-AX task performance

**Fig. 1:**
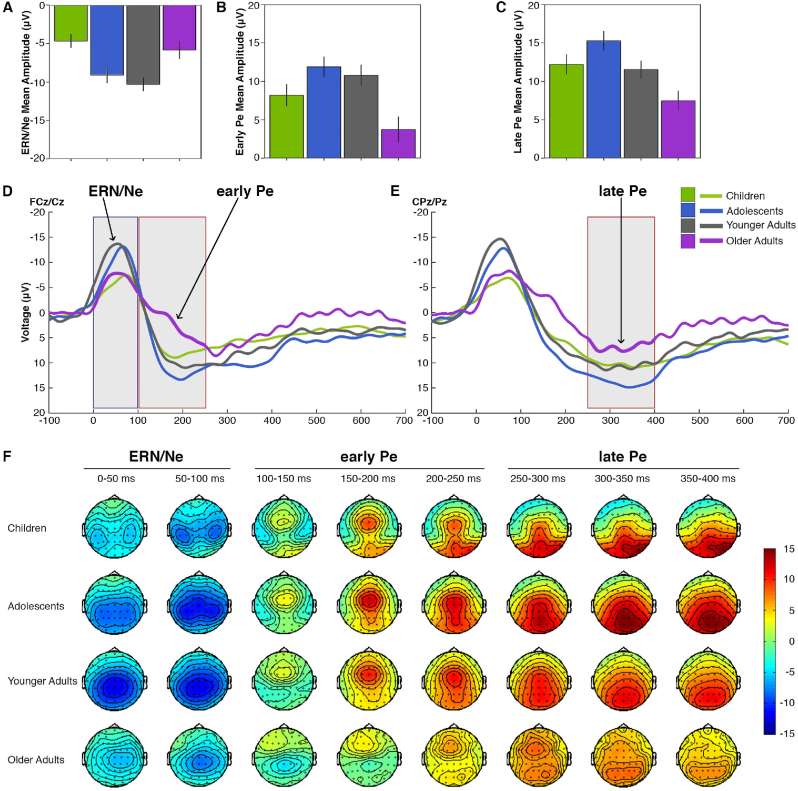
A–E: Grand averages of the response-locked ERP difference waveforms (error – correct) for the four age groups (A–C) and across time (−100 ms before to 700 ms after response, D-E), averaged for electrodes FCz and Cz for the early ERP components (ERN/Ne and early Pe, A-B and D) and for electrodes CPz and Pz for the late Pe component (C, E). Error bars in figures A–C indicate one standard error of the mean (SE). F: Scalp topographies of the response-locked ERP difference waveforms (mean amplitudes) 0–100 ms (ERN/Ne), 100–250 ms (early Pe) and 250–400 ms (late Pe) after response in 50 ms intervals.

Behavioral performance measures (i.e., reaction times (RT) and accuracy) are provided in Table 2. As expected, the analysis of variance of the median reaction time for correct Go responses revealed significant differences between the four age groups (*F* (3,156) = 15.5, *p* < 0.0001, *η*^*2*^ = 0.23; Fig. 1A) with a significant planned linear (*p* = 0.006, *η*^*2*^ = 0.05) and quadratic contrast (*p* < 0.0001, *η*^*2*^ = 0.21) indicating comparable median RTs in adolescents and young adults (*p* = 0.5) and slower median RTs in children and older adults (*p*s ≤ 0.004). Median RTs were slowest in the older adult compared to the children sample (*p* = 0.008). Also, as expected from previous work (Papenberg et al., 2013), RT variability during the CPT-AX task followed a similar U-shaped function across the lifespan (main effect age group: Welch-adjusted *F* (3,85.0) = 36.4, *p* < 0.0001, *η*^*2*^ = 0.49; linear contrast: *p* < 0.0001, *η*^*2*^ = 0.33; quadratic contrast: *p* < 0.0001, *η*^*2*^ = 0.29; see Fig. 1B), with RT variability being largest in the children sample (*p*s < 0.0001) and smallest in young adults (*p*s < 0.02; cf. Papenberg et al., 2013). The frequency of NogoPrime errors declined from childhood to young adulthood (Welch-adjusted *F* (3,85.7) = 25.1, *p* < 0.0001, *η*^*2*^ = 0.36; Fig. 1C). There was no significant difference in accuracy between younger and older adults. Also, since previous analyses in this sample did not find evidence for a within-individual speed-accuracy trade-off across age groups (cf. Hämmerer et al., 2010), we would therefore not assume that age differences in speed-accuracy trade-offs impact on the reported age group differences in ERP components.

**Table 2:** Behavioral Performance of the lifespan sample during the CPT-AX task.

Variable	Children (9–11 years)		Adolescents(13–15 years)		Younger Adults(21–28 years)		Older Adults(66–76 years)	
Go RT median (ms) a	292.6	(39.3)	265.6	(30.4)	270.6	(27.6)	313.4	(37.6)
Go RT variability (ms) a	118.7	(32.7)	70.0	(19.3)	61.7	(14.8)	75.3	(18.0)
NoGoPrime errors (%) a	37.3	(15.7)	24.7	(12.4)	14.8	(9.4)	16.0	(10.6)

*Note.* Values indicate means (*M*) with standard deviations (*SD*) in parenthesis. RT – reaction time.

a Significant age group effects with p < 0.05.

### 3.2 Lifespan differences in ERP correlates of performance monitoring and error awareness

The number of trials of the CPT-AX task that were included into the ERP analysis after EEG artifact correction is provided for each age group in Table 3. Fig. 1 shows the grand averages of the response-locked difference waveforms (Fig. 1A–E) and the scalp topographies (Fig. 1F) for each age group. As can be seen in the scalp topographies (Fig. 1F), peaks reflecting the ERN/Ne, early and late Pe components show a fixed temporal sequence in all four age groups. However, a differential pattern in peak amplitudes across the lifespan was observed in all three ERP amplitudes (Fig. 1A–C). Independent univariate ANOVA models with age group (CH, TE, YA, OA) as between-subject factor were used to test for age group main effects in the three ERP components. Significant age group differences were observed in the ERN/Ne (*F* (3,154) = 8.0, *p* < 0.0001, *η*^*2*^ = 0.13), the early Pe (*F* (3,154) = 6.1, *p* = 0.001, *η*^*2*^ = 0.11), as well as the late Pe amplitude (*F* (3,156) = 6.5, *p* = 0.0004, *η*^*2*^ = 0.11). In contrast, analysis of the ERPs’ latencies showed no age group differences. In a next step, planned contrasts across the four age groups were computed to test for differential lifespan gradients for the three ERPs. The ERN/Ne followed a significant quadratic i.e., inverted U-shaped lifespan pattern (*p* < 0.0001, *η*^*2*^ = 0.12; linear contrast *n. s.*), with a significant increase in amplitude from childhood to adolescents (*p* = 0.001, *η*^*2*^ = 0.07) and a significant decline from young to older adulthood (*p* = 0.002, *η*^*2*^ = 0.06). Specifically, ERN/Ne amplitudes were comparable in adolescents and young adults as well as in children and older adults (*p*s > 0.3). For both Pe components, planned contrasts indicated significant linear (early Pe: *p* = 0.025, *η*^*2*^ = 0.03; late Pe: *p* = 0.001, *η*^*2*^ = 0.06) and quadratic lifespan gradients (early Pe: *p* = 0.0002, *η*^*2*^ = 0.09; late Pe: *p* = 0.004, *η*^*2*^ = 0.05). Whereas early Pe amplitudes were comparable from childhood to young adulthood (*p*s > 0.05), late Pe amplitudes were increased in adolescents compared to young adults (*p* = 0.03, *η*^*2*^ = 0.03). Both Pe components declined in older age compared to the young adulthood (early Pe: *p* = 0.001, *η*^*2*^ = 0.07; late Pe: *p* = 0.003, *η*^*2*^ = 0.03) and the children sample (early Pe: *p* = 0.03, *η*^*2*^ = 0.03; late Pe: *p* = 0.008, *η*^*2*^ = 0.04).

**Table 3:** Number of trials of the CPT-AX task included into the ERP analysis after EEG artifact correction.

Variable	Children	Adolescents	Younger Adults	Older Adults
	(9–11 years)	(13–15 years)	(21–28 years)	(66–76 years)
Go a	218.8 (32.7, 122–263)	231.6 (27.0, 168–265)	244.8 (22.2, 155–268)	246.9 (17.4, 200–268)
NoGoPrime a	22.5 (10.1, 3–46)	16.1 (8.6, 2–33)	9.6 (6.7, 2–27)	8.2 (5.1, 2–21)

*Note.* Values indicate means (*M*) with standard deviations (*SD, range*) in parenthesis.

a Significant age group effects with p < 0.05.

In summary, the ERP results indicate differential lifespan gradients for the ERN/Ne, early Pe and late Pe component of error monitoring and error awareness. The ERN/Ne followed a clear inverted U-shaped pattern of early maturation and aging-related decline. In contrast, both Pe components were attenuated in older age. However, only the late Pe component showed a clear developmental gradient, namely an increase in adolescence, while the early Pe might mature already before the age of 9 years in the present lifespan sample.

### 3.3 Relations between the ERN/Ne and Pe components

Although the ERN/Ne and both Pe components can be temporally dissociated, it has been suggested that the ERN/Ne and the early Pe component share generators and assumed underlying processes of conflict and error detection (Van Veen and Carter, 2002). To further investigate potential relations between the ERN/Ne and the Pe components, we conducted two hierarchical regression models with early or late Pe as dependent variable. Mean effects of age groups, age variability within age groups, as well as preceding ERPs were controlled for by including them as regressors of no interest. As can be seen in Table 4, ERN/Ne amplitude was indeed predictive of the early Pe (*β* = 0.38, *t* = 5.0, *p* < 0.0001; age group main effects for CH: *β* = −0.29, *t* = −3.1, *p* = 0.003 and OA: *β* = −0.42, *t* = −4.7, *p* < 0.0001) but not of the late Pe component (*p* = 0.4). This suggests shared underlying processes for the ERN/Ne and early Pe but does not preclude the possibility of the ERN/Ne and the late Pe component reflecting independent processes. Both the early and late Pe components were significantly correlated in all four age groups (*r*s > 0.5, *p*s ≤ 0.001).

**Table 4:** Hierarchical regression model for ERP predictors.

Model Number	DV	Regressors	Regression results for behavioral predictors after controlling for age and preceding ERPs		
			beta	t-value	p-value
1: ERP	Early Pe	Age group Continuous age ERN/Ne	0.38	4.96	<0.0001
	Late Pe	Age group Continuous age ERN/Ne Early Pe	0.07	0.87	0.388

*Note.* DV – dependent variable; ERN/Ne – error-related negativity; ERP – event-related potential; Pe – error positivity.

### 3.4 Relations between ERP components, behavioral performance, and intra-individual response time variability in the CPT-AX

To further characterize potentially differential processes reflected in the three ERP components, associations between ERP amplitude and behavioral performance measures in the CPT-AX task (i.e., median RT during Go trials, number of NogoPrime errors) were investigated in separate linear hierarchical regression models. As previously, these models controlled for age variability within age groups, mean age group differences and preceding ERP components by including these variables as regressors of no interest before entering median Go-RT (Table 5, model 1) or NogoPrime errors (Table 5, model 2) as regressors into the independent models. We observed no relationship between the ERP components and median RT (see also ERPs sorted by RTs in Fig. S1 in the supplemental material). However, subjects with larger ERN/Ne (*β* = 0.24, *t* = 2.6, *p* = 0.01) as well as larger early Pe amplitudes (*β* = −0.20, *t* = −2.1, *p* = 0.04) showed higher accuracy (i.e., fewer NogoPrime errors). This suggests that ERN/Ne and early Pe amplitudes during error monitoring are not related to inter-individual differences in decision criterions, but rather to inter-individual differences in focusing on correct performance.

**Table 5:** Hierarchical regression models for behavioral predictors (CPT-AX and covariate tasks).

Model Number	DV	Regressors	Regression results for behavioral predictors after controlling for age and preceding ERPs		
			beta	t-value	p-value
2: CPT-AX – Go RT median	ERN/Ne	Age group Continuous age Go RT median	−0.05	−0.57	0.570
	Early Pe	Age group Continuous age ERN/Ne Go RT median	0.11	1.34	0.183
	Late Pe	Age group Continuous age ERN/Ne early Pe Go RT median	−0.07	−0.94	0.349
3: CPT-AX – NogoPrime errors	ERN/Ne	Age group Continuous age NoGoPrime errors	0.24	2.59	0.011
	Early Pe	Age group Continuous age ERN/Ne NoGoPrime errors	−0.26	−2.93	0.004
	Late Pe	Age group Continuous age ERN/Ne Early Pe NoGoPrime errors	0.02	0.24	0.814
4: CPT-AX – intra-individual Go RT variability	ERN/Ne	Age group Continuous age Go RT median RT variability	0.15	1.28	0.204
	Early Pe	Age group Continuous age Go RT median ERN/Ne RT variability	−0.35	−3.37	0.001
	Late Pe	Age group Continuous age Go RT median ERN/Ne Early Pe RT variability	−0.13	−1.38	0.169
5: Cognitive covariate task – inspection time	ERN/Ne	Age group Continuous age Early IT correct	0.03	0.37	0.711
	Early Pe	Age group Continuous age ERN/Ne Early IT correct	0.19	2.52	0.013
	Late Pe	Age group Continuous age ERN/Ne Early Pe Early IT correct	−0.10	−1.52	0.130

*Note.* DV – dependent variable; ERN/Ne – error-related negativity; ERP – event-related potential; IT – inspection time; Pe – error positivity; RT – reaction time. The variable name following the model number in column 1 indicates the regressor that was added into the model for each ERP component after entering the regressors “age group”, “continuous age”, and “preceding ERP”.

Given that intra-individual RT variability during cognitive tasks is assumed to be negatively correlated with DA modulation efficiency (cf. Li and Rieckmann, 2014), we further conducted separate regression models for each of the three ERP components (entered as outcome variables) with the CPT-AX Go-RT variability entered as regressor (Table 5, model 3), which is assumed to be negatively correlated with the efficiency of DA modulation of neuronal gain control (cf. Li and Rieckmann, 2014). Again, all models controlled for age variability, mean age, median RT, and preceding ERP component. We observed larger early Pe amplitudes in individuals with smaller intra-individual RT variability during the CPT-AX task (*β* = −0.35, *t* = −3.8, *p* = 0.001). This is in line with suggestions of insufficient neuronal gain control (e.g., suboptimal tuning of the signal-to-noise ratio in task relevant brain circuitries) in individuals with increased RT variability (cf. Li and Rieckmann, 2014). There were no corresponding effects for the late Pe component and no relations of either ERP component with intra-individual variability of processing speed in the covariate identical pictures task. Regression models are summarized in Table 5 (models 2–4).

### 3.5 Relations between ERP components and covariate measures of early perceptual processing and attention allocation

Finally, to further investigate potential influences of intra-individual variability in early information processing and attention allocation (indicative of DA modulation of cognitive functions), we conducted analogous regression analyses with performance on early inspection time task entered as regressor of interest. Only early Pe amplitudes were (positively) associated with higher accuracy rates during early perceptual processing after controlling for ERN/Ne (i.e., early inspection time; *β* = 0.19, *t* = 2.5, *p* = 0.01; Table 5, model 5).

In summary, both ERN/Ne and early Pe amplitudes were associated with response accuracy, but not response speed during the CTP-AX task. Furthermore, intra-individual response variability during the CPT-AX task was smaller and early visual processing accuracy in the covariate inspection time task was better in subjects with larger early Pe amplitudes.

## 4 Discussion

The present study investigated age differences in ERP correlates of error detection and error awareness in children, adolescents, younger and older adults. While the ERN/Ne has repeatedly been associated with implicit error detection (Di Gregorio et al., 2016; Holroyd and Coles, 2002; Nieuwenhuis et al., 2001; Yeung et al., 2004) and the late Pe with explicit error awareness (Endrass et al., 2007, 2012; Hughes and Yeung, 2011), the role of the early Pe is still unclear in the literature. Planned comparisons across the four age groups revealed distinct patterns of the ERN/Ne as well as the early and late Pe across the lifespan. In line with previous research, all three ERP components were characterized by a marked decline in older age (cf. Hämmerer et al., 2010 for review regarding the ERN/Ne; cf. Hämmerer et al., 2014 for review regarding the ERN/Ne; cf. Band and Kok, 2000; Clawson et al., 2017 regarding the Pe component; Mathewson et al., 2005), suggesting more general effects of aging on the performance monitoring system including deficient focusing of attention during early stimuli processing and inefficient monitoring of erroneous versus correct responses (Hämmerer et al., 2010), presumably as a consequence of aging-related decline in gray and white matter integrity (especially regarding long-range connectivity) and deficient DA modulation of underlying brain circuitries (cf. Hämmerer and Eppinger, 2012; Hämmerer et al., 2014 for reviews). In contrast, maturation-related gradients from childhood to young adulthood differed between the ERN/Ne, the early and the late Pe component.

The ERN/Ne amplitude showed a quadratic increase from childhood to adolescence which, in previous literature, has been related to maturation of microstructure und connectivity of the ACC (e.g., myelinization and synaptic pruning) and increasing efficiency of DA modulation of cortical performance monitoring circuitries (cf. Ferdinand and Kray, 2014 for review). Our finding of an attenuated ERN/Ne in children and in older adults is especially noteworthy given that we did not find evidence for age group differences in speed-accuracy trade-offs. Deficient error detection is therefore not attributable to differences in response strategies between age groups. Moreover, while children committed more errors than younger adults, older adults did not differ in response accuracy from the young adult group, suggesting that the reduced ERN/Ne might be attributable to different evaluation processes in children and older adults (Scheffers and Coles, 2000). Specifically, in children, reduced ERN/Ne amplitudes might be indicative of an error detection system that still lacks the ability to foster accuracy by prioritizing correct over incorrect responses (Falkenstein et al., 2000; Hämmerer et al., 2010, 2014; Segalowitz and Dywan, 2009; Yeung and Cohen, 2006). In contrast, in older adults, a reduced ERN/Ne seems to be less related to fostering correct responses but might rather be indicative of increasing uncertainty about the actual response accuracy, probably due to insufficient selective attention on the relevant cue information (Hämmerer et al., 2014; Scheffers and Coles, 2000; Yeung and Cohen, 2006).

Previous studies investigating maturation and aging effects on the Pe did rarely differentiate the early and late Pe components. Here, we observed no clear maturation effect in children and adolescents as compared to younger adults for the early Pe component. Comparable Pe amplitudes from childhood to young adulthood have been reported before (Davies et al., 2004; Santesso and Segalowitz, 2008; Wiersema et al., 2007), but not specifically for its early component, where evidence is especially sparse. Of note, our results on the early Pe might further indicate that processes related to the early Pe might not necessarily require complete maturation of earlier implicit error detection i.e., of the ERN/Ne (cf. Ferdinand and Kray, 2014). In contrast, the late Pe component was characterized by a significant increase in amplitude in adolescents (13–14 years). A similar age group effect has been observed by Ladouceur et al. (2007), although the authors did not further differentiate the early and the late Pe components. Behaviorally, the adolescent group of the current study showed improved accuracy compared to children but did not yet reach an adult level of performance. However, irrespective of the age group, the late Pe was not related to the number of prime-based errors. Also, the late Pe increase in adolescents was not accompanied by an equivalent increase in the ERN/Ne or early Pe component. The insufficient behavioral post-error adaptation and the late Pe increase in adolescence might therefore be indicative of suboptimal error awareness or subjective overestimation of the actual performance in young adolescents. However, since subjects were not asked to rate their response confidence, potential underlying mechanisms remain speculative.

Aging effects on the Pe components were more consistent in our lifespan study. Similar to the ERN/Ne component, both Pe components were reduced in older adults. Previous research has indicated that (i) the Pe gets larger in amplitude with increasing accumulated evidence for error commission (Steinhauser and Yeung, 2010) and (ii) seems to be related to measures of subjective error awareness (Endrass et al., 2007; Hughes and Yeung, 2011; O'Connell et al., 2007). Our results of reduced Pe amplitudes thus further support the notion that older adults struggle in particular with the accumulation of error evidence during behavioral responses (presumably reflected in an decreased early Pe). This might suggest that older adults are less sure or less aware of having committed an error (presumably reflected in the decreased late Pe), or less able to evaluate errors rapidly (there is for instance evidence for a decline in the attention-related P3 components in older age; Fjell and Walhovd, 2004).

Of particular note, in the given sample we did not observe a slow positivity or negativity shift of the ERN/Ne which superimposed otherwise probably average Pe components. We therefore concluded that the reported Pe results and lifespan gradients are not a mere effect of age group differences in the ERN/Ne.

Analyses of relations of the ERP components with behavioral performance and cognitive covariate measures helped to further qualify as well as distinguish the processes reflected in the ERN/Ne and Pe components. Across all four age groups, response accuracy during the CPT-AX task was larger in subjects with larger ERN/Ne and early Pe amplitudes, which is consistent with empirical evidence indicating that a larger ERN/Ne reflects a stronger focus on correct responses (Danielmeier et al., 2009; Yeung and Cohen, 2006). The relationship between the early Pe and performance accuracy remained significant even after controlling for variance explained by the ERN/Ne amplitudes, suggesting that the early Pe might provide an additional contribution to enabling correct task performance. Interestingly, only the early Pe component was further associated with intra-individual response variability (a potential index of DA modulation of the signal-to-noise ratio of synaptic transmission within cortical networks across the lifespan; cf. Li et al., 2001; Li and Rieckmann, 2014) and early visual processing accuracy (indicative of fast selective attention allocation), indicating smaller RT variability and better processing accuracy in subjects with higher early Pe amplitudes. Differences in functional relations between the early versus late Pe and behavioral measures of inter-individual response variability and attention allocation might also in part indicate that both Pe components are differentially affected by DA modulation, although no direct evidence can be provided without pharmacological DA challenge in the current study.

Since the function of the early Pe component is still largely unclear, our study provides important additional information on the putative role of the early Pe in performance monitoring. The reported link to response accuracy suggests that like the ERN/Ne, the early Pe is implicated in a performance-goal based rapid evaluation of errors (although likely capturing different aspects of a rapid error evaluation given the statistical independence from the ERN/Ne). Moreover, unlike the ERN/Ne, a larger early Pe seems also to be related to lower intra-individual variability of early perceptual processing (RT variability during perceptual processing and accuracy during pre-attentive inspection time i.e., attention allocation performance). This further suggests that the early Pe might be more than just a rebound of the ERN/Ne, which is also supported by the recent finding that the Pe can even be elicited in the absence of the preceding ERN/Ne (Di Gregorio, Maier and Steinhauser, 2018). Our new insights on the role of the early Pe in error monitoring might thus suggest that it reflects an error evaluation process, which is dependent on the content and stability of top-down control on correct responses.

In contrast, the late Pe seems not to be affected by individual differences in ERN/Ne offset but rather showed shared variance with the early Pe. The lack of a relationship between early perceptual processing and the late Pe amplitude further supports the hypothesis that the late Pe is functionally distinct from the ERN/Ne and the early Pe component and indicates that (explicit) error awareness is less affected by noisy perceptual processing, top-down control of correct response tendencies or fast attentional allocation. However, it needs to be taken into account that the late Pe component has a longer oscillation time window than the early Pe component, which might have attenuated a potential similar correlation between the late Pe and RT variability.

Finally, in an earlier examination of stimulus processing during the CPT-AX task across the lifespan (Hämmerer et al., 2010), it was found that conflict perception improves from childhood to young adulthood (i.e., the N2 amplitude following correct Nogo trials assumed to reflect the processing of unexpected stimuli decreases; cf. also Yeung and Cohen, 2006). This presumably indicates a delayed maturation of cognitive control processes such as performance monitoring (e.g., Espinet et al., 2012; Lamm et al., 2006) and underlying brain circuitries including the ACC and dorsolateral PFC (Casey et al., 1997; Jonkman et al., 2007). In old age, however, the magnitude and the distinctiveness of the Go- and the Nogo-N2 was drastically reduced and paralleled by an increase in P3a amplitude after distracting non-cues, suggesting that older adults involuntarily allocated attentional resources to irrelevant non-cues, thereby attenuating precision and efficacy of performance monitoring (Hämmerer et al., 2010). Earlier research also showed differential relations between adult age (20–92 years) and the oddball P3a (reflecting involuntary attention allocation to distractors) versus the P3b (reflecting stimulus evaluation and resource allocation to the target). Relative to the P3b, the P3a was more susceptible to aging-related changes as indicated by a negative correlation between age and P3a amplitude and a negative correlation between age and P3a latency (Fjell and Walhovd, 2004).

These lifespan gradients in the N2 and P3a component are consistent with our current findings on the ERN/Ne and the Pe components, which suggest reduced error awareness or error evaluation in older adults. Taken together, both results suggest potential functional equivalences between stimulus- and response-locked ERP correlates of performance monitoring processes before and during action. More specifically, both the N2 and the ERN/Ne have been linked to ACC-related monitoring functions with the N2 reflecting conflict detection during stimulus processing and response selection, and the ERN/Ne reflecting (implicit) error detection during response execution (Ullsperger et al., 2014 for review; Yeung et al., 2004; Yeung and Cohen, 2006). Similarly, functional parallels have also been assumed for the early/late Pe and the P3a/P3b complexes (cf. Ridderinkhof et al., 2009; Wessel et al., 2011 for review) with the earlier components presumably reflecting fast orientation or (involuntary) fast attention allocation, and the later Pe and P3b components being associated with memory updating. Others observed a strong correlation between the Pe and the P3 component suggesting that the Pe might reflect a P3-like response to the awareness that an error has already occurred (Davies et al., 2001). However, based on our current results and the results of the same task and sample reported by Hämmerer et al. (2010) indicating differing lifespan gradients for the various P3 and Pe components, we cannot conclude that the Pe and the P3 component are equivalent. Rather, the early Pe/P3a and late Pe/P3b components could be considered as functionally distinctive (Polich, 2007; Ullsperger et al., 2014 for review). Of particular note, hierarchical regression analyses in the current study also tentatively support the hypothesis of functional distinctiveness of both Pe components by showing that larger early Pe amplitudes were associated with better early perceptual processing ability and subsequent attention allocation, while no such relationship was found for the late Pe.

To sum up, we cannot entirely exclude potential confounding effects of factors such as reduced synaptic density in the cortex or changes in skull thickness during maturation and aging, but taking the previous analysis of the same sample and task (Hämmerer et al., 2010) as well as previous literature (e.g., Fjell & Walhoved, 2004) into account as control comparisons, our current data indicate that age group differences in ERP components of error monitoring are not uniformly reduced in children and older adults. Instead maturation- and aging-related structural and functional brain changes seem to differentially affect different ERP components and, hence, different error monitoring subprocesses.

### 4.1 Conclusion

By examining performance monitoring in a unique lifespan sample, we observed distinct maturation- and aging-related patterns of the ERN/Ne, the early and the late Pe, which complement the existing knowledge on the development of error detection and error awareness across the lifespan. We showed that while all three ERP components were significantly affected by aging-related decline, only the ERN/Ne showed a clear U-shaped pattern of amplitude increase during maturation and decline in older age. Furthermore, unlike the late Pe, both early components (i.e., the ERN/Ne and the early Pe) were related to behavioral performance accuracy, but only the early Pe seemed affected by early perceptual processing ability and subsequent attention allocation. Accordingly, the following conclusions could be drawn from the present study: First, the late Pe component, assumed to reflect (explicit) error awareness i.e., accumulation of error evidence, seems developmentally and functionally distinct not only from the ERN/Ne but also from the early Pe component. Second, although the early Pe shows greater functional overlap with the ERN/Ne (error detection) than with the late Pe, the early Pe seems not to be a mere rebound effect of the preceding ERN/Ne component but rather reflect complementary aspects of rapid error monitoring such as fast post-error adaptation of selective attention (cf. Maier et al., 2011). Evidence of this study motivates further research exploring the distinct developmental gradients of error signaling and error awareness.

## CRediT authorship contribution statement

**Franka Thurm:** Methodology, Software, Formal analysis, Visualization, Writing - original draft. **Shu-Chen Li:** Conceptualization, Writing - review & editing, Project administration, Funding acquisition, Resources, Supervision. **Dorothea Hämmerer:** Conceptualization, Validation, Methodology, Software, Investigation, Data curation, Writing - review & editing, Supervision.

## Declaration of competing interest

The authors have no actual or potential conflicts of interest.
