# Supplementary material for: Maturation- and aging-related differences in electrophysiological correlates of error detection and error awareness
Source: Neuropsychologia. 2020 Jun;143:107476. doi: 10.1016/j.neuropsychologia.2020.107476 (PMC7322543; doi:10.1016/j.neuropsychologia.2020.107476)
Supplement: Multimedia component 1 [file mmc1.pdf]

Supplemental material

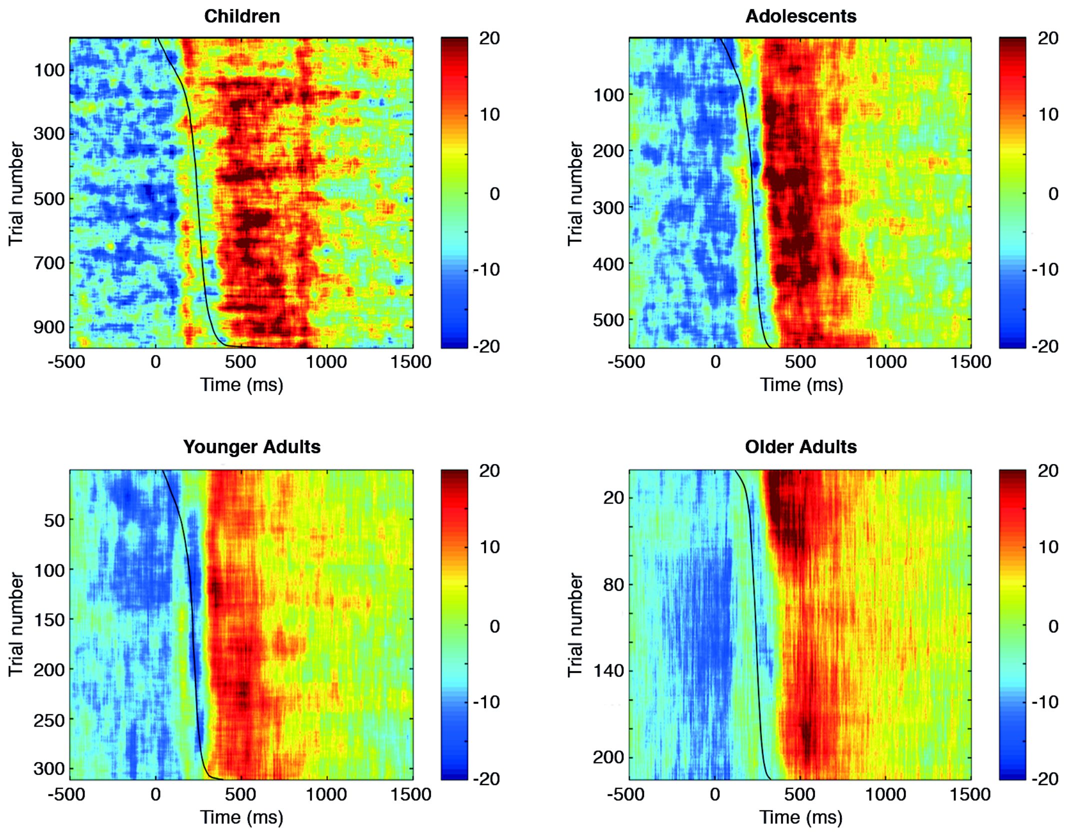

Fig. S1. Relation between ERP mean amplitudes and reaction time (indicated by the black line) across trials (sorted by trial number) and time within trials (-500 ms before to 1500 ms after response).
